# Combining mathematical modeling, *in vitro* data and clinical target expression to support bispecific antibody binding affinity selection: a case example with FAP-4-1BBL

**DOI:** 10.3389/fphar.2024.1472662

**Published:** 2024-10-09

**Authors:** Javier Sanchez, Christina Claus, Christine McIntyre, Tamara Tanos, Axel Boehnke, Lena E. Friberg, Siv Jönsson, Nicolas Frances

**Affiliations:** ^1^ Roche Pharma Research and Early Development (pRED), Roche Innovation Center Basel, Basel, Switzerland; ^2^ Department of Pharmacy, Uppsala University, Uppsala, Sweden; ^3^ Roche Pharma Research and Early Development (pRED), Roche Innovation Center Zurich, Schlieren, Switzerland; ^4^ Roche Pharma Research and Early Development (pRED), Roche Innovation Center Welwyn, Welwyn Garden City, United Kingdom

**Keywords:** immunotherapy, bispecific antibody, modeling, simulation, binding affinity, oncology, pharmacodynamics, FAP-4-1BBL

## Abstract

The majority of bispecific costimulatory antibodies in cancer immunotherapy are capable of exerting tumor-specific T-cell activation by simultaneously engaging both tumor-associated targets and costimulatory receptors expressed by T cells. The amount of trimeric complex formed when the bispecific antibody is bound simultaneously to the T cell receptor and the tumor-associated target follows a bell-shaped curve with increasing bispecific antibody exposure/dose. The shape of the curve is determined by the binding affinities of the bispecific antibody to its two targets and target expression. Here, using the case example of FAP-4-1BBL, a fibroblast activation protein alpha (FAP)-directed 4-1BB (CD137) costimulator, the impact of FAP-binding affinity on trimeric complex formation and pharmacology was explored using mathematical modeling and simulation. We quantified (1) the minimum number of target receptors per cell required to achieve pharmacological effect, (2) the expected coverage of the patient population for 19 different solid tumor indications, and (3) the range of pharmacologically active exposures as a function of FAP-binding affinity. A 10-fold increase in FAP-binding affinity (from a dissociation constant [K_D_] of 0.7 nM–0.07 nM) was predicted to reduce the number of FAP receptors needed to achieve 90% of the maximum pharmacological effect from 13,400 to 4,000. Also, the number of patients with colon cancer that would achieve 90% of the maximum effect would increase from 6% to 39%. In this work, a workflow to select binding affinities for bispecific antibodies that integrates preclinical *in vitro* data, mathematical modeling and simulation, and knowledge on target expression in the patient population, is provided. The early implementation of this approach can increase the probability of success with cancer immunotherapy in clinical development.

## 1 Introduction

**FIGURE 1:**
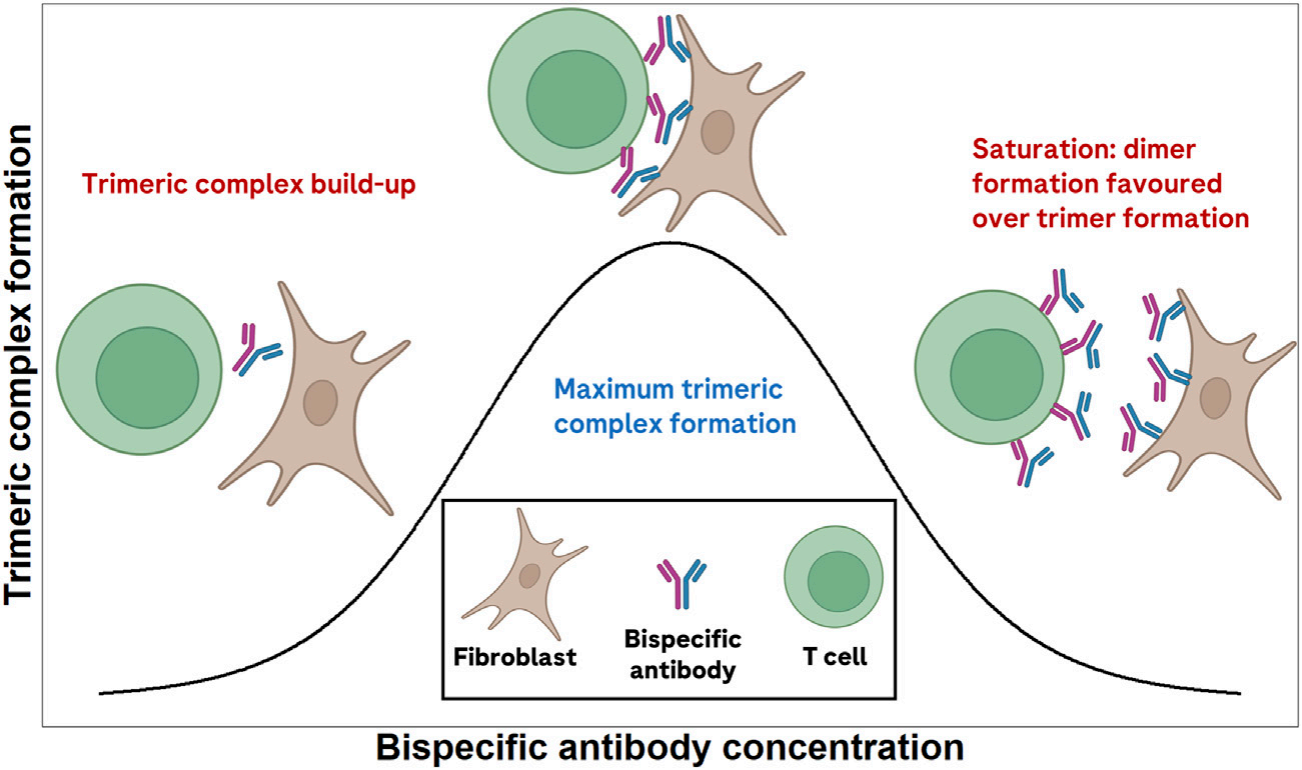
Schematic depiction of the expected bell-shaped curve of trimeric complex formation *versus* bispecific antibody concentration.

Bispecific antibodies in oncology exert their effect by engaging two targets simultaneously (van de Donk and Zweegman, 2023; Brinkmann and Kontermann, 2017). In doing so, they are capable of eliciting tumor cell killing, or tumor-specific T-cell co-stimulation, among others. As such, the pharmacological activity of many of these bispecific antibodies depends on the formation of a so-called trimeric complex entity (the bispecific antibody simultaneously bound to both of its targets). A bell-shaped trimeric complex formation *versus* bispecific antibody concentration (when concentration axis is in log-scale) has been described previously (Betts and van der Graaf, 2020; Douglass et al., 2013; Schropp et al., 2019). Figure 1 schematically depicts the expected trimeric complex *versus* bispecific antibody bell-shaped curve. The shape of this curve is dependent on receptor expression levels (Claus et al., 2023) and the binding affinities of the bispecific to each of its targets (Douglass et al., 2013). When designing a new bispecific antibody, it is relevant to ensure that binding affinities to both receptors are selected to maximize the probability of patient benefit. In doing so, the molecule with the highest *a priori* chance of success can be developed, potentially minimizing the attrition rate of novel cancer immunotherapies.

In order to generate meaningful data in the range of concentrations where maximum trimeric complex formation is expected, the relationship between receptor expression, bispecific antibody concentration, binding affinity and trimeric complex formation needs to be characterized as early as possible during drug development. Mathematical models can be used to incorporate, via ordinary differential equations, the different binding processes of the antibody to its target. In doing so, the relationship between trimeric complex and pharmacological effect can be established and used to make prospective predictions. A workflow combining preclinical *in vitro* experiments with mathematical modeling to characterize such bell-shaped curves has been described (Sanchez et al., 2023).

Next, quantitative knowledge on how receptor expression and binding affinities affect this bell-shaped curve can be used to predict variability in clinical outcomes, both within an indication and across different indications. In turn, this information can be used during the dose-finding stage of clinical development to ensure that a sufficient number of patients per dose cohort are included to select the most relevant dose for pivotal development, as guided by the FDA in the Project Optimus initiative (Shah et al., 2021; Zirkelbach et al., 2022).

To date, several CD3 T-cell bispecific antibodies (eliciting direct tumor killing) have been approved (Dickinson et al., 2022; Budde et al., 2024; Thieblemont et al., 2023; Moreau et al., 2022; Chari et al., 2022). Success with bispecific costimulators (aiming to further activate T cells) has been more elusive despite a rich pipeline of compounds and identified targets, as well as strong preclinical proof-of-concept studies, both *in vitro* and *in vivo* (Claus et al., 2023; Stein et al., 2023; Segal et al., 2023; Shen et al., 2023). The complexity in developing these molecules, including the optimal dose and patient population selection, may have contributed to the lack of clinical success with bispecific costimulators (Claus et al., 2023).

To inform on dose and patient population selection, modeling and simulation approaches have been proposed (Sanchez et al., 2023). In a prior work, we explored the use of *in vitro* data and mathematical modeling to suggest a recommended range of doses to explore in clinical trials when combining the TCB cibisatamab with the bispecific costimulator FAP-4-1BBL. Cibisatamab is a TCB targeting the CEA receptor (expressed in tumor cells) and the CD3 receptor (expressed in T cells) (Bacac et al., 2016). FAP-4-1BBL is a bispecific costimulator targeting the Fibroblast Activation Protein (FAP) expressed in cancer-associated fibroblasts, and the 4-1BB (CD137) receptor, which is expressed in activated T cells (Claus et al., 2019). The combination of cibisatamab and FAP-4-1BBL is being studied in patients with colorectal carcinoma (NCT04826003).

In this work, using a previously published model for a fibroblast activation protein alpha (FAP)-directed 4-1BB (CD137) costimulator (FAP-4-1BBL (RO7122290) (Sanchez et al., 2023), we generate virtual bispecific molecules differing in their FAP-binding affinities (in terms of the dissociation constant, K_D_) to explore the impact of binding affinity on trimeric complex formation and pharmacology. Moreover, the workflow includes the expected FAP variability in the patient population, which is then used to choose the optimal binding affinity to maximize the proportion of patients who can potentially benefit from the therapy.

## 2 Materials and methods

The developed workflow consisted of five different simulation steps. A summary of the aims, endpoints, and conditions used in each simulation is presented in Table 1. Details of the models used for simulations are published elsewhere (Sanchez et al., 2023).

**TABLE 1:** Summary of the different simulations performed.

Simulation aim	Simulated molecules		Bispecific antibody concentration		FAP expression and/or indication	4-1BB expression	
	Two molecules^1^	Range of molecules^2^	Fixed^3^	Variable^4^		Fixed^5^	Variable^6^
Simulation 1: Minimum receptor expression to achieve 90% of maximum pharmacological effect		X	X		Ranging between 100–500,000 FAP/fibroblast, assumed constant over time		X
Simulation 2: Percentage of patient population achieving ≥90% of maximum pharmacological effect	X		X		19 oncology indications (see Zboralski, D. et al) with 5,000 virtual FAP expressions		X
Simulation 3: Bispecific antibody concentrations leading to ≥ 50% of maximum pharmacological effect		X		X	Fixed to median expression in colon cancer (2,560 FAP/cell)		X
Simulation 4: Expected percentage of the maximum pharmacological effect by oncology indication	X		X		19 oncology indications (see Zboralski, D. et al) with 5,000 virtual FAP expressions	X	
Simulation 5: Percentage of the maximum pharmacological effect in clinic at different doses and different schedules	X			X	1,000 virtual FAP expressions (colon cancer indication)	X	

Two molecules^1^: differing in FAP-binding affinity (K_D_, of 0.7 nM or 0.07 nM). Range of molecules^2^: FAP K_D_, values between 0.001 and 10 nM. Fixed^3^: to that resulting in maximum trimeric complex formation for each virtual molecule. Variable^4^: either ranging between 2?10^–5^.

and 50 nM (Simulation 3) or varying over time according to the pharmacokinetic profile of each virtual patient (Simulation 5). Fixed^5^: 150 4-1BB, receptors per T cell. Variable^6^: expression peaks 24–48 h after T-cell bispecific administration, and returns to baseline 72–96 h after treatment start.

### 2.1 Previously conducted experiments and developed models

Details about the experimental setups and model development have been previously described (Sanchez, J. et al., 2023). The conducted experiments aimed to investigate the increase in *vitro* tumor cell killing when combining FAP-4-1BBL with cibisatamab, compared to cibisatamab alone. In brief, the experimental set-up consisted of different *in vitro* cell culture plates with tumor cells (expressing CEA), fibroblasts (expressing different levels of FAP), and peripheral blood mononuclear cells (containing the T cells driving the *in vitro* tumor cell killing). Across different experiments that varied in levels of FAP expression, cibisatamab concentration, or FAP-4-1BBL concentration, both 4-1BB expression as well as *in vitro* tumor cell killing were measured.

Using these data, two mathematical models that are of interest in this manuscript were developed: *Model 1* described, using Ordinary Differential Equations (ODEs), how the number of 4-1BB receptors expressed per T cell changes over time after treatment with cibisatamab with or without FAP-4-1BBL. This model takes into account the 4-1BB expression *versus* cibisatamab concentrations (with higher cibisatamab concentrations leading to higher 4-1BB expressions). 4-1BB expression (measured as number of receptors per T cell) was found to be close to zero at baseline, peaked at around 120–200 receptors per T-cell between 24 and 48 h following cibisatamab stimulation, and returned to baseline 72 h after treatment with cibisatamab.

*Model 2* integrates *Model 1* (describing the changes of 4-1BB expression over time) and describes the dynamic of the trimeric complex formation (Betts et al., 2020) (FAP-4-1BBL bound to both FAP and 4-1BB simultaneously). This model was then used to calculate the number of trimeric complexes formed in each *in vitro* tumor cell killing experiment over a time period of up to 120 h. The relationship between trimeric complex formation and increase in tumor cell killing was found to follow an Emax-like relationship (see Equation 1). A sensitivity analysis of the full model is available in the Supplementary Material.

### 2.2 Software

All simulations were conducted in R version 4.2.1 (R Core Team, 2022) using the rxode2 package (Fidler et al., 2023). Model codes are available in the Supplementary Material.

### 2.3 Target engagement model and trimeric complex formation

Free bispecific antibody was assumed to bind independently and sequentially to the FAP receptor on fibroblasts and 4-1BB receptor on T cells. The binding rate constants of the reference FAP-4-1BBL molecule 4-1BB and FAP receptor were available from *in vitro* experiments (see Supplementary Table S1). The steady-state trimeric complex formation (at the end of a 120 h simulated period) was assumed to be the driver of the pharmacological effect in all simulations except in the clinical simulations (*Simulation 5*). The relationship between the percentage of maximum pharmacological effect and trimeric complex is described by Equation 1 (Sanchez et al., 2023) (see Supplementary Table S1 for parameter values):

$$Pharmacological\;Effect\;\left(\%\;of\;maximum\right)=\frac{100\times {\left(Trimeric\;Complexes\;per\;T\;cell\right)}^{Hill}}{{\left(Trimeric\;Complexes\;per\;T\;cell\right)}^{Hill}+TC_{50}^{Hill}}$$ (1)

where Hill represents the Hill coefficient of the Emax-like relationship, and TC_50_ represents the number of trimeric complexes per T cell required to achieve 50% of the maximum pharmacological effect. The maximum pharmacological effect was derived from *in vitro* data [see (Sanchez et al., 2023)] and corresponds to a 4.4-fold increase in *vitro* tumor cell killing, which was observed when combining FAP-4-1BBL with the CD3 T-cell engager cibisatamab (Bacac et al., 2016). *TC*_*50*_ was estimated to be 3.9 × 10^−2^ trimeric complexes per T cell, and the *Hill* coefficient was estimated to be 1.16. No parameter uncertainty was considered in the simulations.

### 2.4 Virtual molecule generation

Virtual molecules differing in their FAP-binding affinity were generated (FAP K_D_ range between 0.001 and 10 nM). This corresponds to an increase in binding affinity (reduction in K_D_) ranging from 0.07 to 700-fold compared to the original FAP-4-1BBL binding affinity of 0.7 nM (Sanchez et al., 2023). 4-1BB binding affinity was fixed to be equal to that of the reference FAP-4-1BBL molecule (0.2 nM, see Supplementary Table S1). The focus on FAP-binding affinity rather than 4-1BB binding affinity is due to clinical FAP expressions being better quantified than 4-1BB expressions (Zboralski et al., 2022). The relationship between trimeric complex and pharmacological effect was assumed independent from binding affinity.

### 2.5 Simulation of FAP expression in patient populations with different oncology indications

**FIGURE 2:**
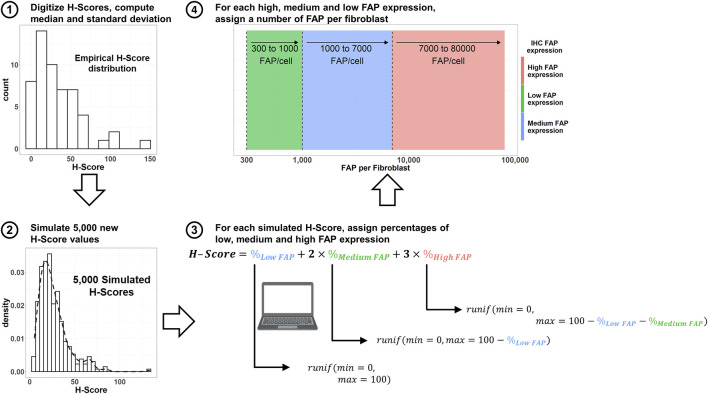
Workflow to simulate both number of FAP per cell, as well as percentage of the tumor plate covered with high, medium and low FAP-expressing fibroblasts using digitized H-Scores from the literature. IHC: immunohistochemistry. FAP: Fibroblast Activation Protein. Runif: random uniform distribution sample.

The workflow for simulation of virtual FAP expressions in patient populations with different solid tumor indications is summarized in Figure 2. FAP H-Scores were retrieved from (Zboralski et al., 2022) by digitization using WebPlotDigitizer (Rohatgi, 2022) for a total of 19 different oncology indications. FAP H-Scores were assumed to be log-normally distributed. The median H-Score was directly scanned from the figure in (Zboralski et al., 2022), while standard deviations of the random effects (omega) were calculated as per Equation 2:

$$\omega =\sqrt{\log\left(1+\frac{sd{\left(x\right)}^{2}}{\bar{x^{2}}}\right)}$$ (2)

where ω represents the standard deviation of the random effects of a log-normal distribution, $sd\left(x\right)$ represents the calculated standard deviation of the scanned H-Scores, and $\bar{x}$ represents the calculated mean of the scanned H-Scores.

In a next step, 5,000 virtual H-Scores per indication were simulated by sampling from a log-normal distribution as per Equation 3:

$$H–Score_{i}=\theta \times e^{\eta_{i}}$$ (3)

where θ represents the scanned median H-Score for that indication and $\eta_{i}$ represents the *i^th^* random sample from a normal distribution with mean equal to zero and standard deviation equal to ω (see Equation 2).

Given that the proportion of high, medium and low FAP areas for each H-Score was not available from (Zboralski et al., 2022), different proportions of high, medium and low FAP-expressing areas satisfying Equation 4 (McCarty et al., 1985) were sampled from a uniform distribution.

$$H–Score=3\times High\;FAP\;area\;\left(\%\right)+2\times Medium\;FAP\;area\;\left(\%\right)+Low\;FAP\;Area\;\left(\%\right)$$ (4)

Finally, a specific number of FAP receptors per fibroblast was associated with the simulated high, medium and low expressing areas by randomly sampling from a uniform distribution. The ranges of FAP expression for such distributions are 300–1,000 (low FAP expression), 1,000–7,000 (medium FAP expression), and 7,000–80,000 (high FAP expression) FAP receptors/fibroblast (Sanchez et al., 2023). From these simulated values, an average number of FAP receptors per fibroblast can be computed and used in the simulations.

### 2.6 General simulation conditions

Unless otherwise stated, the bispecific antibody concentration used across the simulations was the one resulting in maximum trimeric complex formation for each molecule (Douglass et al., 2013) (geometric mean of the simulated FAP-binding affinity for each molecule, and the experimental 0.2 nM binding affinity for the 4-1B binder). 4-1BB change in expression over time was simulated as detailed in (Sanchez et al., 2023) (expression peaks 24–48 h after T-cell bispecific administration, and returns to baseline 72–96 h after treatment start). FAP expression was assumed to not change over time in all simulations. A summary of the conditions used across all simulations is available in Table 1.

### 2.7 Simulation 1: minimum FAP expression per fibroblast to achieve 90% of the maximum pharmacological effect

The simulations were performed under the same conditions as defined for the available *in vitro* data (Sanchez et al., 2023; Claus et al., 2019). In brief, the *in vitro* system conditions included a plate volume of 200 μL, containing 10,000 FAP-expressing fibroblasts (receptor expressions of 92,000 or 12,700 FAP/fibroblast), 5,000 peripheral blood mononuclear cells (PBMCs), and 5,000 MKN-45 tumor cells. To explore the impact of FAP expression in trimeric complex formation and pharmacology, 10,000 FAP-expressing fibroblasts with FAP expressions varying between 100 and 500,000 FAP/fibroblasts were simulated.

### 2.8 Simulation 2: percentage of the patient population achieving at least 90% of maximum effect

Two different bispecific molecules, with FAP-binding affinities of 0.7 nM and 0.07 nM were considered. The minimum number of FAP per fibroblast required to achieve 90% of the maximum pharmacological effect was derived as detailed in *Simulation 1*. Next, 1,000 virtual patients differing in their FAP expression were simulated for each indication (19 in total) as detailed in the section above.

### 2.9 Simulation 3: exposure range to achieve 50% of the maximum effect

FAP expression of fibroblasts was in these simulations fixed to the median expression in the colon cancer indication (2,960 FAP/fibroblast), as colon cancer is an intermediate FAP expression. Bispecific costimulator concentrations ranged between 2 × 10^−5^ and 50 nM.

### 2.10 Simulation 4: expected percentage of maximum pharmacological effect by oncology indication

The percentage of the maximum effect by indication was illustrated using two different molecules (reference molecule, FAP-binding affinity of 0.7 nM, and a 10-fold increase in FAP-binding affinity molecule, i.e., K_D_ of 0.07 nM). 4-1BB expression was fixed to be constant at 150 4-1BB receptors per T cell.

### 2.11 Simulation 5: percentage of the maximum pharmacological effect in the clinic at different doses and different schedules

Pharmacokinetic/Pharmacodynamic (PK/PD) simulations in the clinical conditions were conducted for the reference FAP-4-1BBL molecule and for a virtual molecule with a 10-fold increase in FAP-binding affinity *versus* the reference molecule. 1,000 virtual colon cancer patients differing in both pharmacokinetic (PK) parameters and FAP expressions were created. Parameter values from the 2-compartment model describing FAP-4-1BBL PK (see Supplementary Table S1) were available from the literature (Micallef et al., 2020). PK was assumed to be independent of FAP-binding affinity for the main analysis. In an alternative analysis (Supplementary Material), the increased FAP-binding affinity was assumed to increase target-mediated drug disposition (TMDD). The bispecific antibody tumor distribution was in the model described with a first-order tumor uptake model (without inter-individual variability) resulting in an approximate 2.2:1 plasma:tumor distribution ratio (Eigenmann et al., 2021).

Different scenarios combining three different schedules (once a week [qw], once every 2 weeks [q2w], and once every 3 weeks [q3w]) and 40 different doses (ranging from 1 to 150 mg) were simulated. In the simulations, the two molecules were administered as a 2 h infusion to the central compartment. Trimeric complex formation in the tumor was linked to the percentage of the maximum benefit as per Equation 1. To evaluate the best doses and schedules for each virtual molecule, the average percentage of the maximum pharmacological benefit from the time of the initial infusion up to 42 days (representing a total of 6, 4, or 2 administrations for a qw, q2w and q3w schedule) was used. Supplementary Figure S1 summarizes the workflow followed in the clinical simulations.

## 3 Results

### 3.1 Impact of FAP-binding affinity on trimeric complex-exposure relationship

**FIGURE 3:**
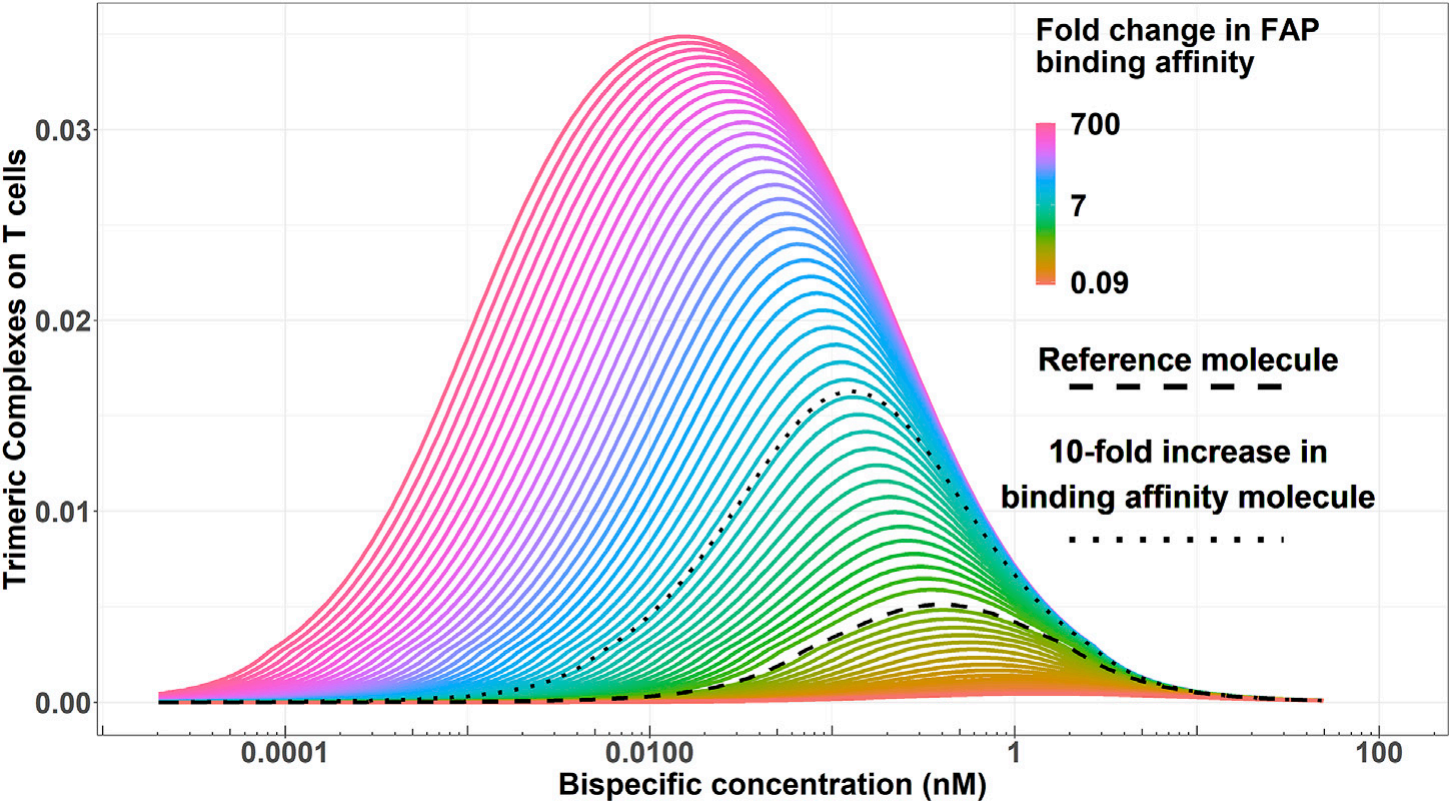
Simulated trimeric complex formation for each virtual bispecific antibody molecule (varying in their FAP-binding affinity) for a given range of concentrations. Color codes represent the FAP-binding affinity. The dashed black line highlights the reference molecule. The dotted black line highlights an enhanced binding affinity molecule with a 10-fold increase in FAP-binding affinity *versus* the reference molecule.

Figure 3 displays the simulated trimeric complex formation in a given range of concentrations for each of the generated virtual molecules. An increased binding affinity on the FAP receptor resulted in an increased trimeric complex formation regardless of the bispecific antibody exposure and a decrease in the concentration needed to achieve the maximum of the trimeric complex formation.

### 3.2 Simulations 1 and 2: minimum FAP expression per fibroblast to achieve 90% of the maximum pharmacological effect and percentage of patients achieving this level

**FIGURE 4:**
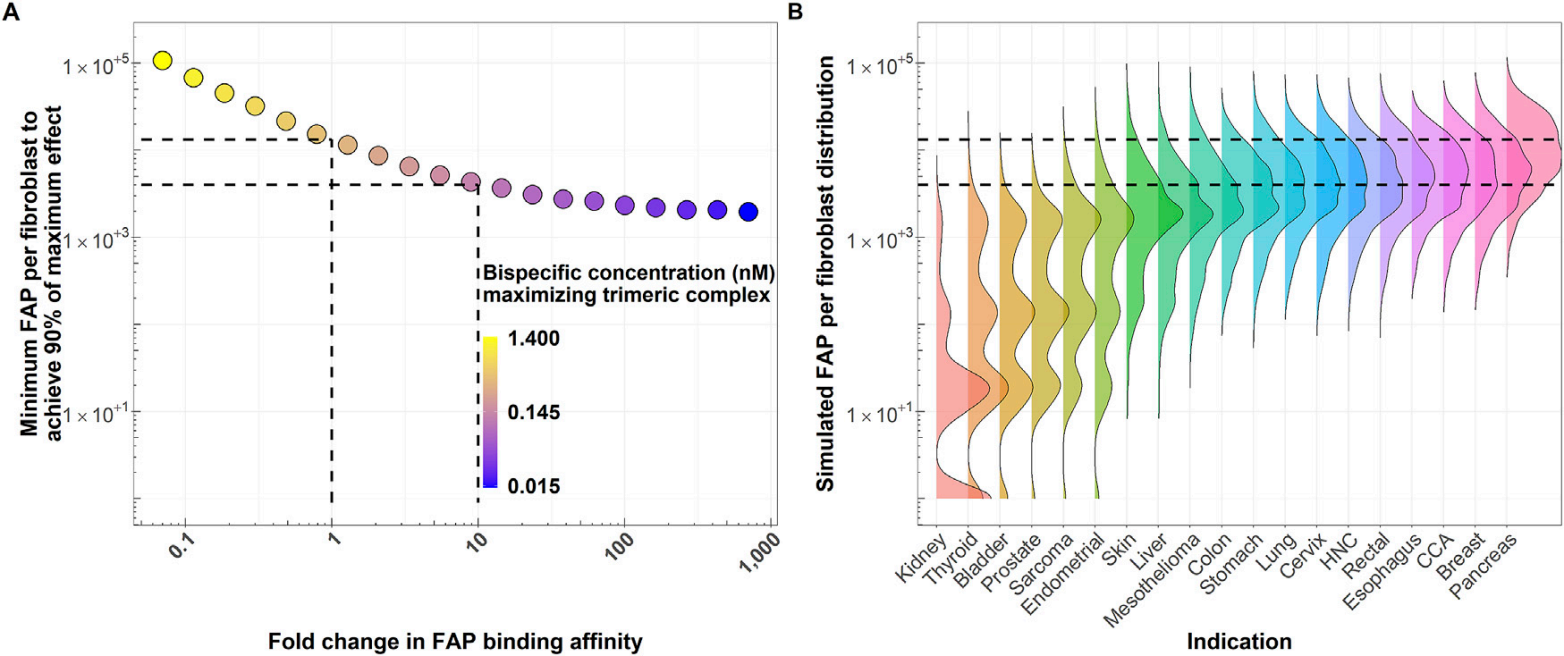
**(A)** FAP receptors per fibroblasts required to achieve at least 90% of the maximum pharmacological effect as a function of fold change in FAP-binding affinity of the bispecific costimulator (see Simulation 2). Dashed lines highlight the reference molecule (FAP K_D_ of 0.7 nM) and a molecule with 10-fold increase in FAP-binding affinity, together with their associated minimum FAP per fibroblast required to achieve 90% of the maximum pharmacological effect. **(B)** Simulated average number of FAP receptors per fibroblast distributions across different solid tumor indications (right). A total of 5,000 expressions per indication are simulated as detailed in Figure 2. Horizontal dashed lines highlight the minimum FAP expression required to achieve 90% of the maximum pharmacological effect with the current molecule (FAP-binding affinity: 0.7 nM) and a molecule with 10-fold increase in FAP-binding affinity. These thresholds are derived from the simulations depicted in panel **(A)**. HNC: Head and neck cancer. CCA: Cholangiocarcinoma.

Figure 4A shows how the number of FAP receptors required to achieve 90% of the pharmacological effect decreases when increasing the FAP-binding affinity of the bispecific costimulator. The number of FAP receptors to achieve 90% of the pharmacological effect decreases from 13,400 to 4,000 receptors when the FAP-binding affinity increases 10-fold. This increase in FAP affinity leads (Figure 4B) to a higher percentage of the patient population expected to benefit from this type of treatment. For an intermediate FAP expression indication, such as colon cancer (median H-Score: 20), 6% of the patient population would achieve at least 90% of the maximum pharmacological effect with the reference molecule, whereas 39% would achieve this effect with a molecule with a 10-fold increase in FAP-binding affinity. The percentage of patients expected to achieve 90% of the maximum pharmacological effect for the two molecules (FAP K_D_ of 0.7 and 0.07 nM) and all indications is available in Table 2. At low FAP expressions (for instance, kidney cancer), the simulated FAP expression distribution appears multimodal (with modes representing the zero, low, medium and high FAP expression). More granular data (percentages of no, low, medium and high FAP expression in tumors) would better recapitulate the FAP expression in this patient population.

**TABLE 2:** Percentage of patients expected to achieve 90% of the maximum pharmacological effect per indication and molecule.

Indication	Original molecule (FAP K_D_ 0.7 nM) (%)	Enhanced affinity molecule (FAP K_D_ 0.07 nM) (%)
Kidney	0	0.2
Thyroid	0	1.4
Bladder	0.1	1.4
Prostate	0	2
Sarcoma	0.2	5
Endometrial	1	8.9
Skin	3.9	22.8
Liver	3.4	24
Mesothelioma	6.3	33
Colon	5.6	39.4
Stomach	9.1	44.8
Lung	9.8	49.4
Cervix	11.8	49.8
HNC	10.2	49.7
Rectal	14.2	54.7
Esophagus	14.4	58.5
CCA	15.2	57.4
Breast	16.7	59.5
Pancreas	36.4	79.3

HNC: head and neck cancer; CCA: cholangiocarcinoma.

### 3.3 Simulation 3: exposure range leading to at least 50% of the maximum pharmacological effect as a function of FAP-binding affinity

**FIGURE 5:**
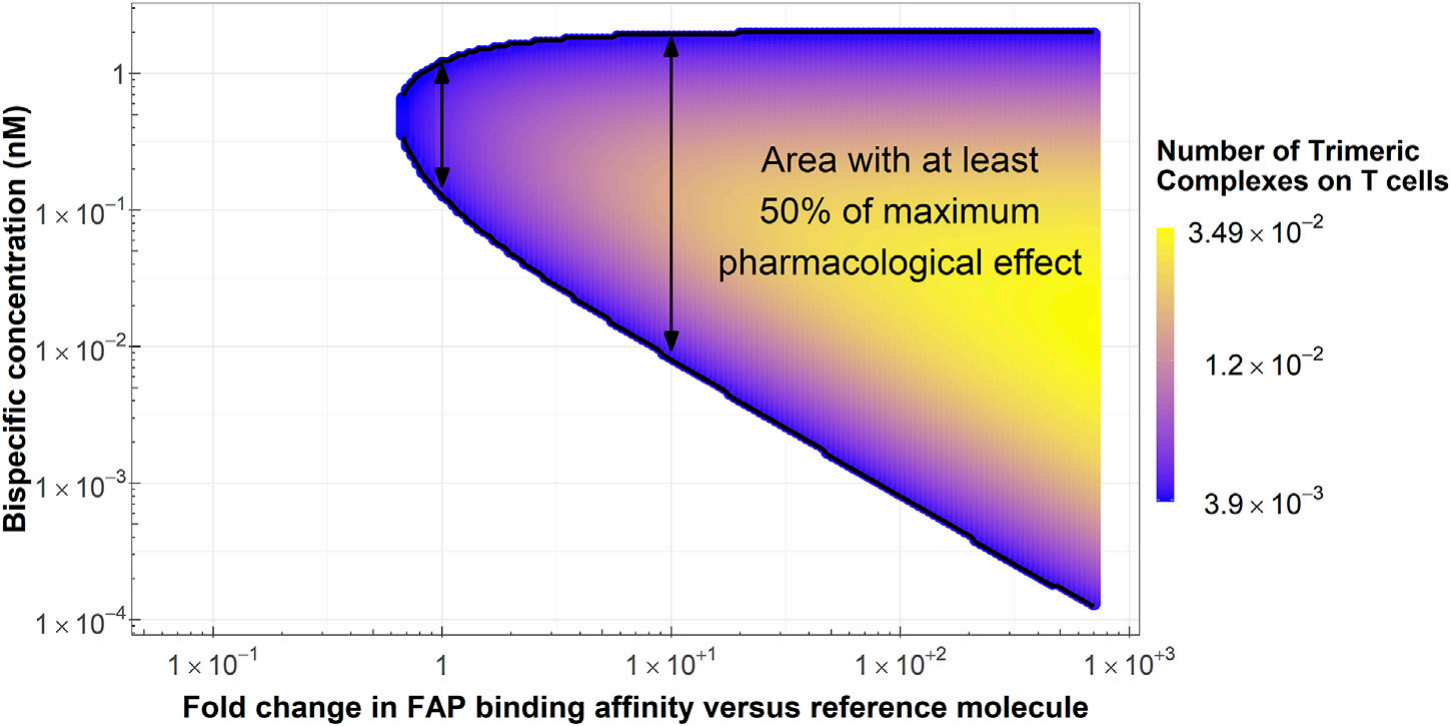
Conditions in terms of bispecific antibody concentration and FAP-binding affinity to achieve at least 50% of the maximum pharmacological effect at the median colon cancer FAP expression. Arrows highlight the range of bispecific antibody concentrations where at least 50% of the maximum pharmacological effect is achieved with the reference molecule (left arrow) and a molecule with a 10-fold increase in FAP-binding affinity (right arrow).

The colored area in Figure 5 represents scenarios where at least 50% of the maximum pharmacological effect is predicted to be achieved as a function of FAP-binding affinity and bispecific antibody concentration for the typical colon cancer FAP expression (2,960 FAP/fibroblast). The range of bispecific antibody exposures leading to 50% of the maximum pharmacological effect increases with increasing FAP-binding affinity. As an illustration, at least 50% of the maximum pharmacological effect can be achieved in a concentration range between 0.1 and 1.2 nM with the reference molecule, compared to a range of 0.01–2 nM for a molecule with a 10-fold higher binding affinity on the FAP receptor. This increase in bispecific antibody range exposure is due to more trimeric complexes being formed at increased FAP-binding affinities, as denoted in Figure 5. Of note, at the concentration maximizing trimeric complex formation for each molecule, the amount of trimeric complexes formed per T cell would be 0.005 for the reference molecule and 0.016 for the molecule with a 10-fold increase in FAP-binding affinity (3.2-fold increase). Lastly, molecules with FAP-binding affinities 1.7-fold lower than that of the reference molecule are unable to achieve 50% of the maximum pharmacological effect regardless of bispecific antibody exposure.

### 3.4 Simulation 4: percentage of maximum pharmacological effect for each indication

**FIGURE 6:**
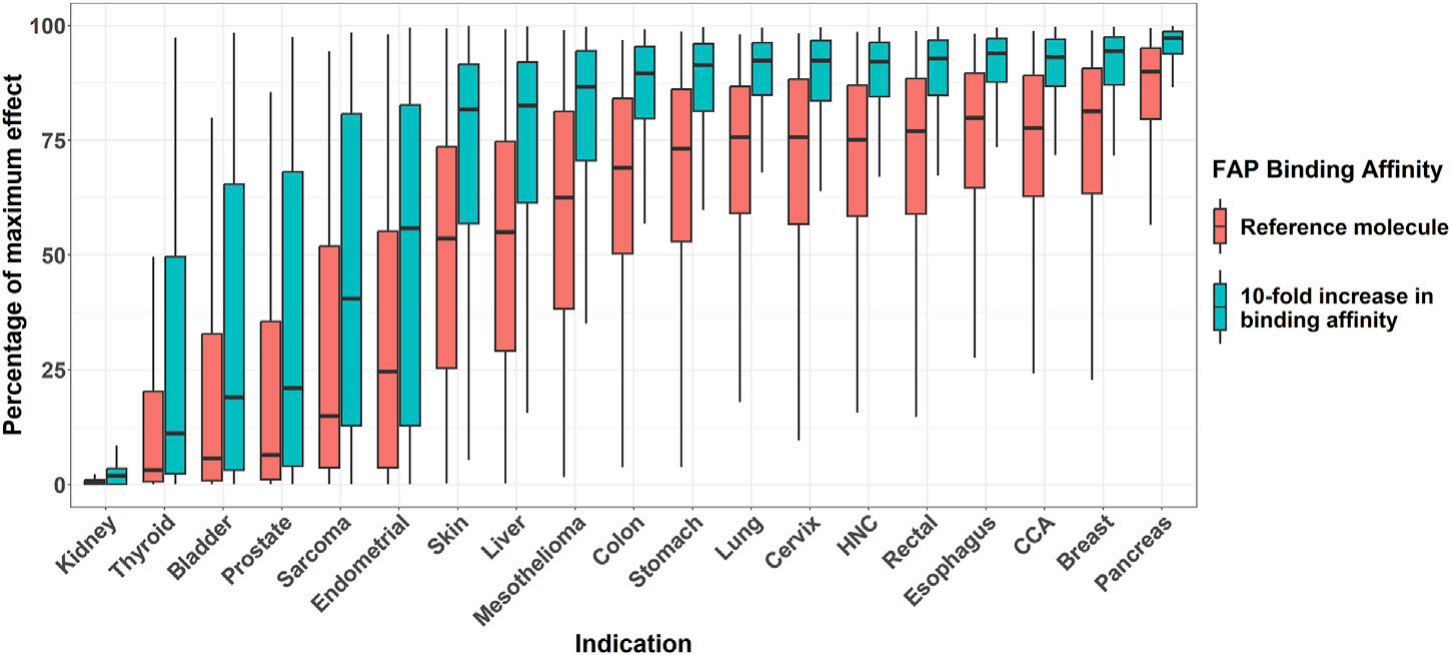
Boxplots of effect (percentage of maximum) across different solid tumor indications for two virtual molecules differing in their FAP-binding affinity. Bispecific antibody concentration is fixed, for each molecule, to the one resulting in maximum pharmacological effect. Mid-solid line represents median, hinges represent 25th-75th percentiles, and upper and lower whiskers extend to the largest and smallest observation which is less than 1.5 interquartile distance from its hinge, respectively. HNC: head and neck cancer. CCA: cholangiocarcinoma.

Figure 6 shows the percentage of the maximum pharmacological effect distribution across different solid tumor indications in a 1,000 virtual patient population differing only in FAP expression. It can be noted how, across all indications, the virtual bispecific costimulator with 10-fold higher FAP-binding affinity *versus* the reference molecule results in an increase in the percentage of the maximum pharmacological effect achievable in all indications. The largest increase in percent of the maximum pharmacological effect was found for the endometrial cancer indication (median pharmacological effect of 24.6% and 55.8% for the reference and the enhanced binding affinity molecules, respectively).

### 3.5 Simulation 5: percentage of the maximum pharmacological effect in the clinic at different doses and schedules

**FIGURE 7:**
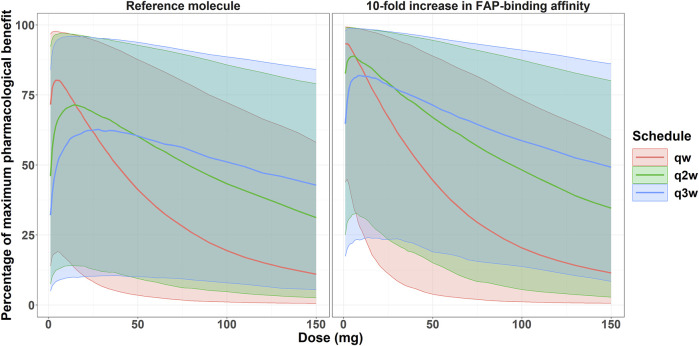
Expected percentage of the maximum pharmacological effect *versus* costimulator bispecific administration protocol in the clinical setting for the colon cancer indication. Solid lines represent the median of 1,000 virtual patients treated at different doses and three different schedules. Shaded areas represent the 5th-95th percentiles of the 1,000 virtual patient population.

Figure 7 depicts the expected percentage of the maximum pharmacological effect expected at different doses and schedules for 1,000 virtual patients (differing both in PK parameters and in FAP expression) treated with the reference bispecific costimulator molecule or a molecule with a 10-fold increase in FAP-binding affinity. The simulations are conducted for the colon cancer indication (intermediate to high FAP expression [median H-Score of 20]). Further simulations assuming increased TMDD with the enhanced affinity molecule and for low FAP (bladder cancer) and high FAP (esophagus) indications are available in the Supplementary Material (Supplementary Figure S2). From Figure 7, it can be noted how the enhanced affinity molecule resulted in a higher percentage of the maximum pharmacological effect across all doses and schedules. Derived from the simulations depicted in Figure 7, Table 3 summarizes the doses resulting in the largest median percent of the maximum pharmacological effect for each schedule and molecule.

**TABLE 3:** Summary of doses maximizing the percentage of the maximum pharmacological effect for each molecule and schedule.

	Reference molecule		Molecule with 10-fold increase in FAP-binding affinity	
Schedule	Dose maximizing pharmacological effect (mg) [cumulative dose over 42 days]	Median percentage of the maximum effect (5th-95th percentile)	Dose maximizing pharmacological effect (mg) [cumulative dose over 42 days]	Median percentage of the maximum effect (5th-95th percentile)
qw	4 [24]	80.2 (18.6–97.7)	2 [12]	93.3 (44.9–99.2)
q2w	14 [42]	71.4 (14.0–96.7)	6 [18]	88.9 (32.4–99)
q3w	28 [56]	62.7 (10.4–95.3)	9 [18]	81.9 (23.2–98.6)

With the reference molecule, the percent of the maximum pharmacological effect was 80% (at the qw schedule) for the virtual patient population with colon cancer indication, compared to 93% with the molecule with a 10-fold increase in FAP-binding affinity. Furthermore, the enhanced binding affinity molecule still achieved a median maximum benefit of 82% at the q3w dosing schedule, highlighting the potential for less frequent dosing schedules. Conversely, the current molecule at the q3w schedule achieved a maximum pharmacological effect of 63%. Importantly, only one scenario (4 mg at a qw schedule) would allow a median percent of the maximum pharmacological effect of 80% with the reference molecule, whereas doses between 1 and 22 mg, both at qw, q2w, and q3w schedules, would allow a median benefit greater or equal to 80% with the molecule with a 10-fold increase in FAP-binding affinity.

## 4 Discussion

For bispecific costimulators similar to FAP-4-1BBL (Claus et al., 2019; Link et al., 2018; Warmuth et al., 2021), an increase in FAP-binding affinity is expected to increase the percentage of patients that can achieve 90% of the maximum pharmacological benefit. As an illustration, with a 10-fold increase in FAP-binding affinity *versus* the reference FAP-4-1BBL molecule, the percentage of patients achieving at least 90% of the maximum pharmacological benefit would be expected to increase from 6% to 39% in the colon cancer indication. In the clinical setting, the predicted median percentage of the maximum pharmacological effect would increase from 80% with the reference molecule to 93% when increasing the FAP-binding affinity by ten-fold. In addition, the range of doses and schedules at which at least 80% of the maximum pharmacological benefit can be achieved is also expanded with increases in FAP-binding affinity. These results highlight the potential of a model-based approach to the affinity selection of bispecific antibodies similar to FAP-4-1BBL.

Despite both a strong biological rationale and promising preclinical data (Claus et al., 2019; Muik et al., 2022b; Warmuth et al., 2021; Sum et al., 2021), no bispecific costimulatory molecules have to date been approved by regulatory authorities. The development of these therapeutic modalities is challenging, but the integration of experimental data and mathematical modeling has the potential to guide both the design of optimized molecules and a refined clinical development strategy. The relationship between preclinical *in vitro* data exploring the change in pharmacological effect *versus* target expression at different bispecific antibody concentrations can be described in mathematical models. From here, the relationship between trimeric complex and pharmacological effect can be inferred, and the binding affinity can be selected to maximize the likelihood of achieving the desired pharmacological effect at a given target expression level. Thorough preclinical characterization of bispecific molecules differing in their binding affinity has earlier been conducted (Warmuth et al., 2021), albeit without being guided by modeling and simulation. Our approach aims to reduce the number of candidate molecules and the number of test conditions required for their evaluation, as well as to select the best molecule candidate and to increase the chance of success for those molecules.

We used the case example of the bispecific costimulator FAP-4-1BBL and explored *in silico* the impact of FAP-binding affinity on pharmacological effect *versus* dose/exposure as well as expected patient response. This work highlights how with bispecific antibodies like FAP-4-1BBL the highest possible doses do not necessarily need to be explored in clinical trials, focusing instead on exploring doses where trimeric complex formation (and, in consequence, pharmacological benefit) is expected to be maximized Our results suggest that, in the case of FAP-directed 4-1BB costimulators, an increase in FAP-binding affinity would decrease the FAP expression threshold to achieve pharmacological benefit, compared to a molecule previously tested in phase I studies (Melero et al., 2023). Furthermore, we found that the expected range of exposures at which at least 50% of the maximum effect could be achieved increased notably (more than 16-fold) with this ten-fold increase in FAP-binding affinity. In practice, there is a higher chance to hit the target exposure clinically with the enhanced molecule than with the reference molecule. In addition, across the range of exposures resulting in at least 50% of the maximum effect, the enhanced molecule will always form more trimeric complexes than the reference molecule, potentially leading to a higher pharmacological effect.

Across the clinical simulations, the high variability in the percentage of maximum pharmacological effect reflects the variability of FAP expression in the patient population. As a result, identifying the optimal dose based solely on the emerging clinical data from small patient cohorts may be challenging. Therefore, selecting molecules expected to form more trimeric complexes across a wider range of exposures has the potential to simplify early clinical development. Furthermore, the indications with the highest *a priori* level of target expression can be prioritized in a rational way, as this workflow can provide expression thresholds to guide indication inclusion, increasing the chances of success. The presented approach has the potential to change how candidate molecules are selected, from selection being based mostly on the results of preclinical experiments, to an approach that designs the molecule with the highest probability of success given the indications of interest. To date, however, there are no clinical data available to validate this approach.

Results from this work suggests that a molecule with a higher FAP-binding affinity would always be beneficial, in terms of increased trimeric complex formation leading to a higher pharmacological effect. In the clinical setting, it needs to be excluded that a less favorable PK (e.g., increased FAP-binding affinity resulting in higher TMDD) would not impair this benefit.

Similar experiments to the ones used in our modeling framework are frequently performed for candidate molecule selection. With our approach, the candidate molecule design can be informed by *in silico* modeling, rather than solely based on the results of the early preclinical experiments. In doing so, the choice of binding affinity can be informed by the target expression in the patient population of interest, prioritizing the molecules with the highest chance of clinical success. Frequently, the selection of the best candidate for a bispecific costimulator is guided by preclinical *in vivo* experiments where each candidate is tested at similar doses. Then, the molecule with the highest effect is selected for further development. However, given that trimeric complex formation is maximized at different doses depending on the binding affinity, lead molecule selection is not always straightforward. Our workflow can ensure that the first proof-of-concept *in vivo* studies use doses resulting in exposures that maximize trimeric complex formation for each candidate molecule, as the bell-shaped curve can be anticipated solely from the binding affinities. Other variability sources, such as tumor uptake or T-cell infiltration in tumors, are not accounted for in this work. In the presented clinical simulations, a plasma:tumor distribution ratio of 2.2:1 was assumed, although this parameter value requires clinical confirmation. Furthermore, we assume that the added benefit of FAP-4-1BBL is independent from the TCB used as a combination partner. In reality, however, it is likely that not the same TCB can be used as a combination partner across all indications. In consequence, the exact level of contribution of FAP-4-1BBL to the effect needs to be elucidated on a case-by-case basis.

Across the model simulations, the FAP-binding affinity was varied while keeping the 4-1BB binding affinity constant. This work can be expanded to include the effect of 4-1BB binding affinity on expected pharmacology with more information on clinical 4-1BB expression variability and change in 4-1BB expression over time. The relationship between trimeric complex and pharmacology was assumed molecule-independent. Given that it is the 4-1BB stimulation what drives the effect, whether this assumption holds when varying 4-1BB affinities would require further confirmation. Furthermore, the work was conducted using average FAP expression per fibroblast. In reality, a heterogeneous FAP expression within tumors is expected. Given that 4-1BB expression following T cell activation is low (about 150 receptors per T cell), the predicted number of trimeric complexes across the different tested scenarios is also low (below one trimeric complex per T cell). These numbers are lower than what has been described with TCBs (where CD3 expression is between 50,000 and 100,000 receptors per T cell (Carpentier et al., 2009; Bikoue et al., 1996)). However, this number appears sufficient to trigger a pharmacological effect in the experimental conditions of the *in vitro* system used to develop the model (Claus et al., 2019; Sanchez et al., 2023).

Finally, the proposed workflow aims to optimize the efficacy from bispecific co-stimulators. FAP is considered a relatively clean target, as its expression is restricted to the tumor microenvironment and tumor draining lymph nodes (Juillerat-Jeanneret et al., 2017; Denton et al., 2014), with little to no on-target-off-tumor toxicity expected as demonstrated in clinical trials with FAP-4-1BBL (Melero et al., 2023). The use of other tumor-associated targets, such as HER2 (Shen et al., 2023), PD-L1 (Warmuth et al., 2021; Muik et al., 2022b) or CD40 (Muik et al., 2022a) may require a more extensive safety profiling. A similar analysis relating trimeric complex to a safety marker (*e.g.*, cytokine release) could be integrated in the workflow. Off-target effects (4-1BB costimulation as a result of binding to antigens other than FAP) was not considered in the proposed workflow.

## Conclusion

Our modeling and simulation workflow suggests that increased binding affinity on FAP would allow for refined molecule design with a FAP-4-1BBL bispecific co-stimulator. A 10-fold increase in binding affinity compared to a molecule already tested in clinic (Melero et al., 2023) is already expected to result in pharmacological benefit over the first generation FAP-4-1BBL molecule. This workflow can be refined for other molecules and targets, if the adequate *in vitro* data is generated to support model development.

## Data Availability

The original contributions presented in the study are included in the article/Supplementary Material, further inquiries can be directed to the corresponding author.
