# Supplementary material for: Combining mathematical modeling, in vitro data and clinical target expression to support bispecific antibody binding affinity selection: a case example with FAP-4-1BBL
Source: Front Pharmacol. 2024 Oct 9;15:1472662. doi: 10.3389/fphar.2024.1472662 (PMC11497128; doi:10.3389/fphar.2024.1472662)
Supplement: Supplementary file 1 [file DataSheet2.docx]

################################################################################

##### THIS SCRIPT REPRODUCES THE WORKFLOW CONDUCTED IN THE MANUSCRIPT ####

##### TO BE ABLE TO EXECUTE IT, YOU MAY NEED SOME PACKAGES AND TO ####

##### INPUT SOME VALUES ####

################################################################################

set.seed(3210) #Optional seed for reproducibility

# This packages are required:

library(MASS); library(dplyr) ; library(rxode2) ; library(ggplot2) ; library(cowplot)

## Model set-up: this is the full model assuming constant 4-1BB expression ----

Model_constant_41BB <- rxode2({

Cc_FAP41BBL = C_FAP41BBL/(V1/1000)

d/dt(C_FAP41BBL) = -CL/V1 * C_FAP41BBL - Q/V1 * C_FAP41BBL + Q/V2 * P_FAP41BBL - Vmax * Cc_FAP41BBL/(Cc_FAP41BBL + Km)

d/dt(P_FAP41BBL) = Q/V1 * C_FAP41BBL - Q/V2 * P_FAP41BBL

d/dt(Tumor_FAP41BBL) = kain * Cc_FAP41BBL - kout * Tumor_FAP41BBL

Cc_FAP41BBL_nM = Cc_FAP41BBL/0.177

Tumor_FAP41BBL_nM = Tumor_FAP41BBL/0.177

R_nM(0) = Tumor_cells_number * E_T_ratio/100 * Four1BB_per_Tcell * 1e9/6.022e23 * 1/Tumor_volume_L#Initial amt in nM of 41BB

d/dt(FAP_41BB_free) = + koff_FAP * FAP_41BB_FAP + koff_41BB * FAP_41BB_41BB - kon_FAP * FAP * FAP_41BB_free -kon_41BB * R_nM* FAP_41BB_free +

kain * Cc_FAP41BBL_nM - kout * FAP_41BB_free

d/dt(FAP_41BB_FAP) = kon_FAP * FAP * FAP_41BB_free + koff_41BB * Trimeric_Complex - kon_41BB * R_nM* FAP_41BB_FAP -koff_FAP * FAP_41BB_FAP

d/dt(FAP_41BB_41BB) = kon_41BB * R_nM* FAP_41BB_free + koff_FAP * Trimeric_Complex - kon_FAP * FAP * FAP_41BB_41BB - koff_41BB * FAP_41BB_41BB

d/dt(Trimeric_Complex) = kon_41BB * R_nM* FAP_41BB_FAP + kon_FAP * FAP * FAP_41BB_41BB - koff_FAP * Trimeric_Complex - koff_41BB * Trimeric_Complex

FAP_0 = Tumor_cells_number * Median_FAP_per_Fibroblast * Median_FAP_pos/100 * 1e9/6.022e23 * 1/Tumor_volume_L * Proportion_Fibroblasts_in_tumor

FAP(0) = FAP_0

d/dt(FAP) = koff_FAP * Trimeric_Complex + koff_FAP * FAP_41BB_FAP - kon_FAP * FAP * FAP_41BB_free - kon_FAP * FAP * FAP_41BB_41BB

d/dt(R_nM) = koff_41BB * Trimeric_Complex + koff_41BB * FAP_41BB_41BB - kon_41BB * R_nM * FAP_41BB_free - kon_41BB * R_nM * FAP_41BB_FAP

RO_FAP = ((FAP_41BB_FAP + Trimeric_Complex)/(FAP_41BB_FAP + Trimeric_Complex + FAP)) * 100 #ReceptoR_nMoccupancy foR_nMFAP (%)

Synapses_on_FAP = (Trimeric_Complex/(FAP + Trimeric_Complex + FAP_41BB_FAP)) * 100

RO_41BB = (Trimeric_Complex + FAP_41BB_41BB)/(Trimeric_Complex + R_nM+ FAP_41BB_41BB) * 100

Total_41BB = Trimeric_Complex + R_nM + FAP_41BB_41BB

Synapses_41BB = Trimeric_Complex/Total_41BB * Four1BB_per_Tcell

Max_benefit = (Emax - Emin) * ((Synapses_41BB)^Hill)/(Synapses_41BB^Hill + EC50^Hill) + Emin

})

## Simulation of FAP expressions from H-Scores ----

#Step 1: input what is the mean, median and standard deviation of the H-Scores:

Mean_H_Scores <- 11 # User input is required; 13 as default

Median_H_Scores <- 10 # User input is required; 10 as default

Std_dev_H_Scores <- 4 # User input is required; 4 as default

#Step 2: compute omega:

Omega_H_Scores <- sqrt(log(1 + (Std_dev_H_Scores**2)/(Mean_H_Scores)**2))

#Step 3: set thresholds for low, medium and high FAP-expressing areas:

Min_value <- 300

Threshold_medium <- 1000

Threshold_high <- 7000

Max_value <- 80000

#Step 4: select how many replicates (virtual expressions/patients) to simulate:

Sample_size_FAP_expressions <- 1000 # User input is required; 1000 as default

#Step 5: simulate virtual H-Scores with computed omega and provided median:

Simulated_H_Scores <- Median_H_Scores * exp(rnorm(Sample_size_FAP_expressions,

mean = 0,

sd = Omega_H_Scores))

#Step 6: round H-Scores to nearest integer (H-Scores are integer-bound):

Simulated_H_Scores <- round(Simulated_H_Scores)

#Step 6: initialize empty vectors to store simulated values:

Prop_High <- vector()

Prop_Medium <- vector()

Prop_Low <- vector()

#Step 7: create a loop to simulate percentages of FAP-positive tumor areas

#compatible with the H-Score we want to reproduce:

for(i in 1:Sample_size_FAP_expressions){

Weight_i <- runif(n = 1, min = 1, max = 3)

Reproduce_H_i <- Simulated_H_Scores[i] #For which H-Score will we compute FAP expression?

# If H-Scores are zero or one, special workflow:

if(Reproduce_H_i == 0){ #If H-Score is zero, all expressions are zero

Prop_High_i <- 0 ; Prop_Medium_i <- 0 ; Prop_Low_i <- 0

} else if(Reproduce_H_i == 1){

#If H-Score is 1, only possibility is 1% of tumor with low expression

Prop_High_i <- 0 ; Prop_Medium_i <- 0 ; Prop_Low_i <- 1

} else{ #If H-Scores are not zero and not one, multiple possibilities exist:

#Propose percentages whose sum does not exceed 100:

Prop_first_i <- runif(1, 0, 100)

Prop_second_i <- runif(1, 0, 100 - Prop_first_i)

Prop_third_i <- 100 - Prop_second_i - Prop_first_i

Collect_Proposal <- c(Prop_first_i, Prop_second_i, Prop_third_i)

#Which of the proposed percentages will be high (1), medium (2), low (3)?

Randomized_order <- sample(x = 1:3, size = 3, replace = F)

#Proposed percentages assigned to their respective areas:

Prop_High_i <- Collect_Proposal[Randomized_order[1]]

Prop_Medium_i <- Collect_Proposal[Randomized_order[2]]

Prop_Low_i <- Collect_Proposal[Randomized_order[3]]

# What H-Score corresponds to these areas?

Propose_H_Score_i <- 3 * Prop_High_i + 2 * Prop_Medium_i + Prop_Low_i

# As long as we don't find proportionswhich match what we need, we keep iterating:

while(round(Propose_H_Score_i) != Reproduce_H_i){

#Weights are assigned beforehand, it helps with convergence at low H-Score values

#by forcing % of FAP-positive areas to be lower

Prop_first_i <- runif(1, 0, min(100, Reproduce_H_i/Weight_i))

Prop_second_i <- runif(1, 0, min(100, Reproduce_H_i/Weight_i) - Prop_first_i)

#Prop_third_i <- runif(1, 0, 100 - Prop_second_i - Prop_first_i)

Prop_third_i <- min(100, Reproduce_H_i/Weight_i) - Prop_second_i - Prop_first_i

Collect_Proposal <- c(Prop_first_i, Prop_second_i, Prop_third_i)

Randomized_order <- sample(1:3, 3, F)

Prop_High_i <- Collect_Proposal[Randomized_order[1]]

Prop_Medium_i <- Collect_Proposal[Randomized_order[3]]

Prop_Low_i <- Collect_Proposal[Randomized_order[2]]

Propose_H_Score_i <- 3 * round(Prop_High_i) + 2 * round(Prop_Medium_i) + round(Prop_Low_i)

} #Close while loop

} #Close else statement

#Once suitable values are found, while loop is exited and values are collected:

#Concatenate values to previously created vector:

Prop_High <- c(Prop_High, round(Prop_High_i))

Prop_Low <- c(Prop_Low, round(Prop_Low_i))

Prop_Medium <- c(Prop_Medium, round(Prop_Medium_i))

}

#Step 8: create a dataframe containing the simulated H-Scores and their

#associated FAP-positive (low, medium and high intensity) areas:

FAP_Expressions <- data.frame(H_Scores = Simulated_H_Scores) %>%

# Add proportions

mutate(Proportion_High = Prop_High,

Proportion_Medium = Prop_Medium,

Proportion_Low = Prop_Low) %>%

#Step 9: for each area (low, medium, high expressing), assign randomly what

#the number of FAP per cell is:

mutate(Seq_low = round(runif(n = Sample_size_FAP_expressions,

min = Min_value, max = Threshold_medium)),

Seq_medium = round(runif(n = Sample_size_FAP_expressions,

min = Threshold_medium, max = Threshold_high)),

Seq_High = round(runif(n = Sample_size_FAP_expressions,

min = Threshold_high, max = Max_value))) %>%

#What is the total FAP positive area?

mutate(FAP_pos_area = Proportion_High + Proportion_Medium + Proportion_Low) %>%

#What is the average FAP expression per fibroblast?

mutate(Average_FAP = Seq_High * Proportion_High/100 + Seq_medium * Proportion_Medium/100 + Seq_low * Proportion_Low/100)

## Model parameters:----

#Number of virtual patients to simulate (best is to match it to

#number of virtual FAP expressions simulated later)

Virtual_pts_number <- 1000 #User input required ; default 1000

#From multivariate normal distributions for parameters which are correlated

mvdistr <- MASS::mvrnorm(n = Virtual_pts_number, mu = c(0, 0), Sigma = matrix(c(0.306, 0.365, 0.365, 0.798), 2, 2)) #1st col is Vmax, 2nd is Km

Vmax_IIV <- mvdistr[, 1] ; Km_IIV <- mvdistr[, 2]

mvdistr <- MASS::mvrnorm(n = Virtual_pts_number, mu = c(0, 0), Sigma = matrix(c(0.485, 0.04, 0.04, 0.104), 2, 2)) #1st col is V1, 2nd is CL

V1_IIV <- mvdistr[, 1] ; CL_IIV <- mvdistr[, 2]

PK_parameters_typical <- c(V1 = 3770, V2 = 830, Q = 21.9,

CL = 15.4, Vm = 0.455, Km = 5.18)

PK_parameters_IIV <- data.frame(V1 = 3770 * exp(V1_IIV),

V2 = 830 * exp(rnorm(Virtual_pts_number, 0, sqrt(1.52))),

Q = 21.9, #No variability on Q

CL = 15.4 * exp(CL_IIV),

Vm = 0.455 * exp(Vmax_IIV),

Km = 5.18* exp(Km_IIV))

# Other model parameters:

Tumor_volume_L <- (4/3 * pi * 25 * 25/3 * 25/3)/1e6 #Volume of ellipsoid with longest diameter = 50 mm, and other diameters = Longest/3

Params_TC_model_IIV <- data.frame(koff_41BB = 0.1, kon_41BB = 0.5, kon_FAP = 0.1/0.7, koff_FAP = 0.1, Four1BB_per_Tcell = 150,

E_T_ratio = 0.1, Tumor_cells_number = 1e7 * Tumor_volume_L * 1000, #Assumption from "Does the cell number 10^9 still really fit one gram of tumor tissue?", and transformation of volume from liters back to cm^3

Tcell_percentage_in_tumor = 40, Tumor_area_covered_FAP = 100, FAP_per_Fibroblast = 92000, Proportion_Fibroblasts_in_tumor = 0.15,

Tumor_volume_L = Tumor_volume_L, Median_FAP_per_Fibroblast = FAP_Expressions$Average_FAP, #Will be replaced once values are simulated

Median_FAP_pos = FAP_Expressions$FAP_pos_area,

Emax = 100, EC50 = 0.003812303, Emin = 0,

Hill = 1.164655,

################## FAP41BBL parameters ######################

V1 = PK_parameters_IIV$V1,

V2 = PK_parameters_IIV$V2,

CL = PK_parameters_IIV$CL,

Q = PK_parameters_IIV$Q,

Vmax = PK_parameters_IIV$Vm,

Km = PK_parameters_IIV$Km, #h for time, mL for vol, ug(/mL) for MM clearance

###### Tumor uptake #######

kain = 0.03 * 2, #2.2 fold distribution plasma:tumor

kout = 0.137)

##Clinical simulations as an example; user can adjust what to simulate----

#Step 1: Define dosing parameters (dose, schedule, addl):

Dose <- 20 #mg

Dosing_schedule <- 3 #weeks

Total_doses <- 10

#Step 2: simulation time, simulation granularity

Max_sim_time <- 30 #weeks

Step <- 0.25 #Step, IN HOURS!!!!!!!!

#Dosing overrides max sim time

Simulation_length <- max(Max_sim_time * 7 * 24,

(Total_doses + 1) * Dosing_schedule * 7 * 24)

#Step 3: create event table for simulation:

Event_information <- et() %>%

add.dosing(dose = Dose, dosing.interval = Dosing_schedule * 7 * 24, #hours

nbr.doses = Total_doses) %>%

add.sampling(time = seq(0, Simulation_length, by = Step))

#Step 4: run the simulation

out <- rxSolve(object = Model_constant_41BB,

params = Params_TC_model_IIV,

events = Event_information)

##Plotting results of simulations----

#Summarize output:

Summarized_out <- out %>% group_by(time) %>%

summarize( # Medians

Median_Exposure = median(Cc_FAP41BBL),

Median_TCs = median(Synapses_41BB),

Median_Benefit = median(Max_benefit),

# Percentiles:

Exposure_PI_low = quantile(Cc_FAP41BBL, probs = 0.05),

Exposure_PI_high = quantile(Cc_FAP41BBL, probs = 0.95),

TCs_PI_low = quantile(Synapses_41BB, probs = 0.05),

TCs_PI_high = quantile(Synapses_41BB, probs = 0.95),

Benefit_PI_low = quantile(Max_benefit, probs = 0.05),

Benefit_PI_high = quantile(Max_benefit, probs = 0.95))

#Very simple summary plot:

Exposure_plot <- ggplot(Summarized_out, aes(x = time/(24*7))) +

geom_ribbon(aes(ymax = Exposure_PI_high,

ymin = Exposure_PI_low),

fill = "lightblue", alpha = 0.6) +

geom_line(aes(y = Median_Exposure), linewidth = 1.25) +

scale_y_log10() + annotation_logticks(base = 10, sides = "l") +

theme_bw() + xlab("Time (weeks)") + ylab("Bispecific plasma concentration (mg/L)")

TCs_plot <- ggplot(Summarized_out, aes(x = time/(24*7))) +

geom_ribbon(aes(ymax = TCs_PI_high,

ymin = TCs_PI_low),

fill = "lightblue", alpha = 0.6) +

geom_line(aes(y = Median_TCs), linewidth = 1.25) +

scale_y_log10() + annotation_logticks(base = 10, sides = "l") +

theme_bw() + xlab("Time (weeks)") + ylab("Trimeric Complexes")

Benefit_plot <- ggplot(Summarized_out, aes(x = time/(24*7))) +

geom_ribbon(aes(ymax = Benefit_PI_high,

ymin = Benefit_PI_low),

fill = "lightblue", alpha = 0.6) +

geom_line(aes(y = Median_Benefit), linewidth = 1.25) +

theme_bw() + xlab("Time (weeks)") + ylab("Pharmacological benefit (%)")

#Arrange plots:

cowplot::plot_grid(Exposure_plot, TCs_plot, Benefit_plot, align = "v", nrow = 1)
