# Supplementary material for: Combining mathematical modeling, in vitro data and clinical target expression to support bispecific antibody binding affinity selection: a case example with FAP-4-1BBL
Source: Front Pharmacol. 2024 Oct 9;15:1472662. doi: 10.3389/fphar.2024.1472662 (PMC11497128; doi:10.3389/fphar.2024.1472662)
Supplement: Supplementary file 2 [file DataSheet1.docx]

Combining Mathematical Modeling, *in vitro* data and clinical target expression to support bispecific antibody binding affinity selection: a case example with FAP-4-1BBL

Javier Sanchez^1,2*^, Christina Claus^3^, Christine McIntyre^4^, Tamara Tanos^1^, Axel Boehnke^1^, Lena E. Friberg^2^, Siv Jönsson^2^, Nicolas Frances^1^

^1^Roche Pharma Research and Early Development (pRED), Roche Innovation Center Basel, Basel, Switzerland

^2^Department of Pharmacy, Uppsala University, Uppsala, Sweden

^3^Roche Pharma Research and Early Development (pRED), Roche Innovation Center Zurich, Schlieren, Switzerland

^4^Roche Pharma Research and Early Development, Roche Innovation Center Welwyn, Welwyn Garden City, UK

**Correspondence:**Corresponding Author
javier.sanchez_fernandez@roche.com

***Supplementary Materials and Methods***

*Table S1. Full model parameters used in the different simulations, as well as source for the value. RSE: relative standard error.*

| ***Parameter (units)*** | ***Description*** | ***Value (% RSE)*** | ***Source*** |
| --- | --- | --- | --- |
| ***FAP-4-1BBL binding properties*** | | | |
| *K_D, FAP_ (nM)* | *Equilibrium dissociation constant for FAP-4-1BBL binding to FAP* | *0.7* | *In vitro binding experiments (data not shown) Parameter is varied during simulations* |
| *k_off, FAP_ (h^-1^)* | *Dissociation rate constant between FAP-4-1BBL and FAP* | *0.1* | *Assumed* |
| *k_on, FAP_ (nM^-1^∙h^-1^)* | *Association rate constant between FAP-4-1BBL and FAP* | *0.143* | *Calculated as k_off_/K_D_. Parameter is varied during simulations* |
| *K_D, 4-1BB_ (nM)* | *Equilibrium dissociation constant for FAP-4-1BBL binding to 4-1BB* | *0.2* | *In vitro binding experiments (data not shown)* |
| *k_off, 4-1BB_ (h^-1^)* | *Dissociation rate constant between FAP-4-1BBL and 4-1BB* | *0.1* | *Assumed* |
| *k_on, FAP_  (nM^-1^∙h^-1^)* | *Association rate constant between FAP-4-1BBL and 4-1BB* | *0.5* | *Calculated as k_off_/K_D_* |
| ***FAP-4-1BBL PK and tumor distribution*** | | | |
| *V1 (mL)* | *Central compartment volume of distribution for FAP-4-1BBL* | *3770 (2.4)* | *Micallef, S. et al. (2020)* |
| *V2 (mL)* | *Peripheral compartment volume of distribution for FAP-4-1BBL* | *830 (3.0)* |  |
| *CL (mL/h)* | *Elimination clearance for FAP-4-1BBL* | *15.4 (3.9)* |  |
| *Q (mL/h)* | *Inter-compartmental clearance for FAP-4-1BBL* | *21.9 (6.3)* |  |
| *Vmax (µg/h)* | *Maximum elimination rate for nonlinear elimination of FAP-4-1BBL* | *0.455 (5.6)* |  |
| *K_M_ (µg/mL)* | *Michaelis-Menten constant for nonlinear elimination of FAP-4-1BBL* | *5.18 (7.1)* |  |
| *k_ain_ (h^-1^)* | *First-order distribution rate for FAP-4-1BBL from plasma to tumor* | *0.06 (—)* | *Assumed, leading to 2.2:1 plasma:tumor distribution ratio* |
| *k_aout_ (h^-1^)* | *First-order distribution rate for FAP-4-1BBL from tumor to plasma* | *0.137 (—)* |  |
| ***Change in 4-1BB receptor expression over time as a function of cibisatamab and FAP-4-1BBL concentration*** | | | |
| *k_trans_ (h^-1^)* | *Transit rate constant* | *0.280 (5.2)* | *Estimated in Monolix2021R1 using in vitro data* |
| *k_out_ (h^-1^)* | *4-1BB elimination rate* | *0.283 (19)* |  |
| *Emax (receptors/cell)* | *Maximum achievable Signal value for cibisatamab* | *537 (13)* |  |
| *Cibisatamab_50, Signal_ (nM)* | *Cibisatamab concentration at which Signal value is half of Emax* | *16 (19)* |  |
| *Hill_Expression_ (—)* | *Hill coefficient for Signal as a function of cibisatamab concentration* | *0.210 (34)* |  |
| *k_in_ (receptors/cell ∙ h)* | *Basal 4-1BB synthesis rate* | *2.95 (21)* |  |
| *Signal2 (receptors/cell)* | *Signal in cibisatamab 20 nM + FAP-4-1BBL 1 nM condition* | *382 (28)* |  |
| *Stop_time_ (h)* | *Time after which Signal value is 0* | *16.2 (19)* |  |
| ***Fold increase in tumor cell killing with respect to cibisatamab monotherapy as a function of trimeric complex formation with FAP-4-1BBL*** | | | |
| *Trimerics_50_ (trimerics on 4-1BB per T cell)* | *Number of trimeric complexes per T cell at which fold increase in tumor cell killing is half of Emax* | *3.9×10^-3^ (17)* | *Estimated in R v4.2.1 using in vitro data* |
| *Hill_Kill, Combination_ (—)* | *Hill coefficient for fold increase in tumor cell killing as a function of trimeric  complex formation equation* | *1.16 (17)* |  |


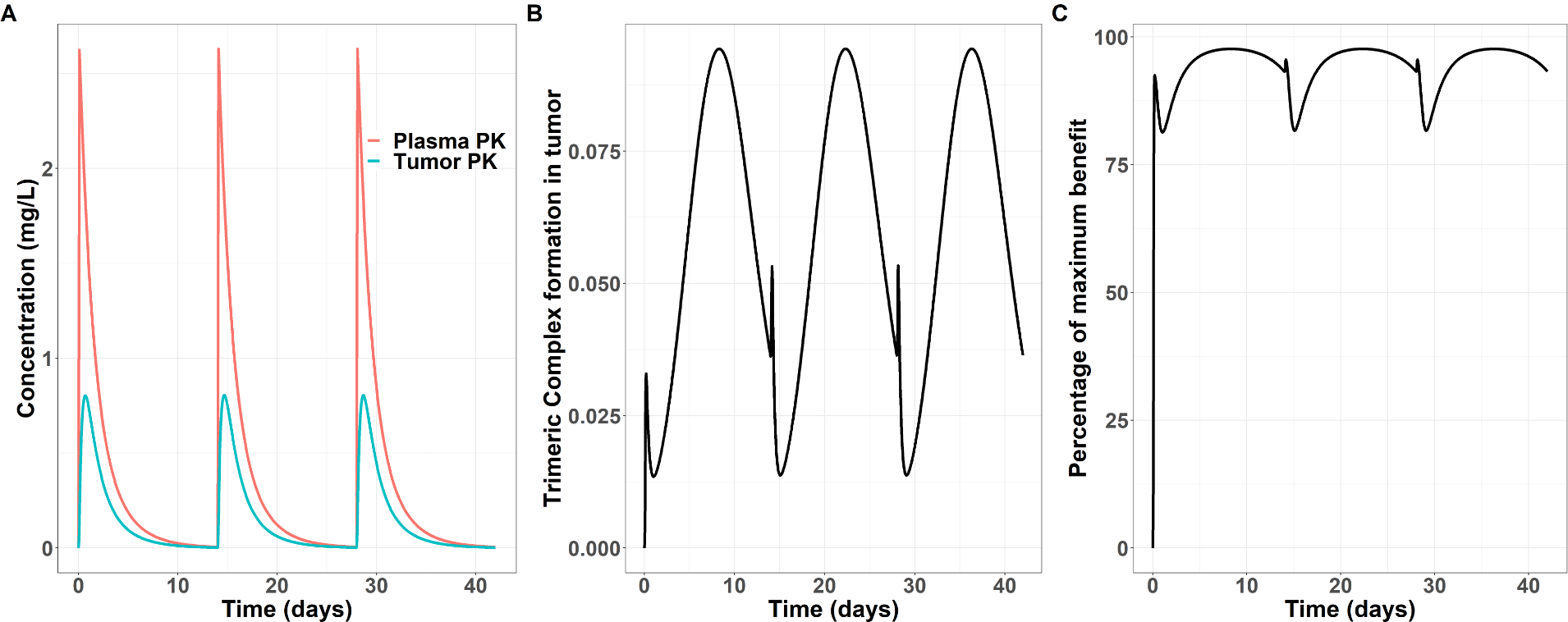
 *Figure S1. Schematic representation of the workflow followed to simulate the expected clinical benefit at a given dose, for a given schedule, for a virtual patient. A, plasma PK and tumor distribution. B, trimeric complex formation. C, associated percentage of the maximum benefit over the considered treatment period.*

**
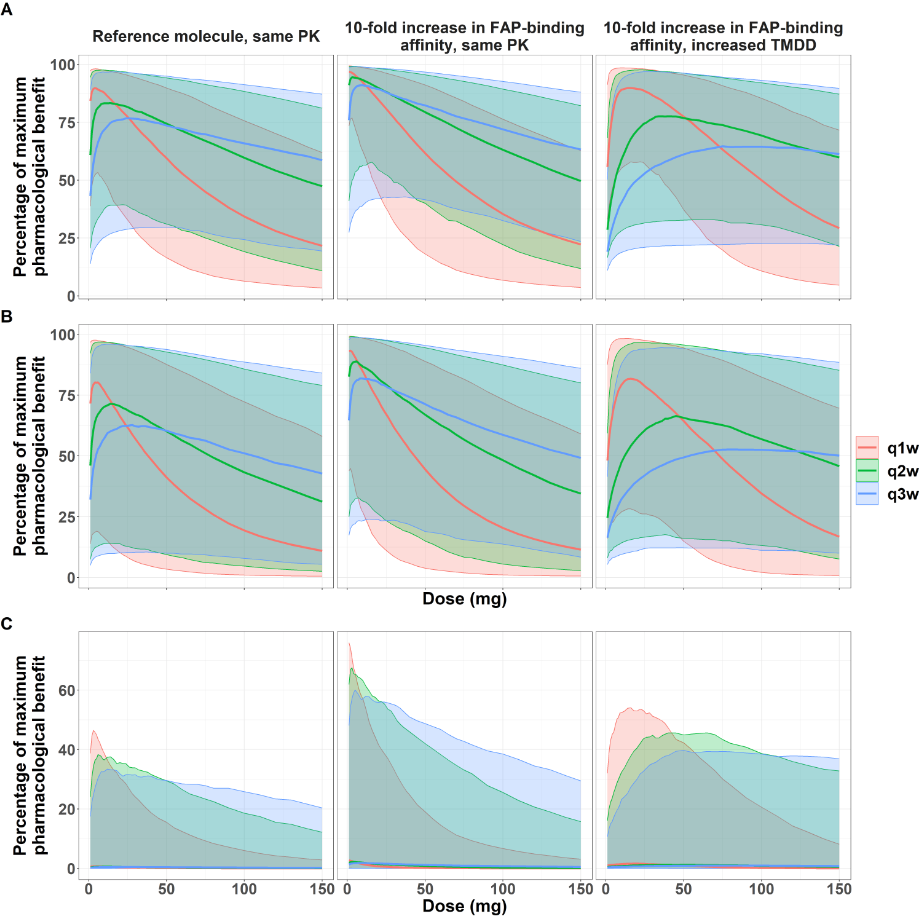
**

*Figure S2. Expected effect at different doses and different schedules for two different molecules varying in FAP-binding affinity, assuming identical PK regardless of FAP-binding affinity (middle panels) or 10-fold increase in TMDD (right-most panels). The increased TMDD scenario is simulated by assuming a reduction in Km equal to the reduction in the FAP K_D_. Rows depict three different indications: Esophagus cancer (high FAP, panel* ***A****), colon cancer (intermediate FAP expression, panel* ***B****) and bladder cancer (low FAP expression, panel* ***C****).*

*
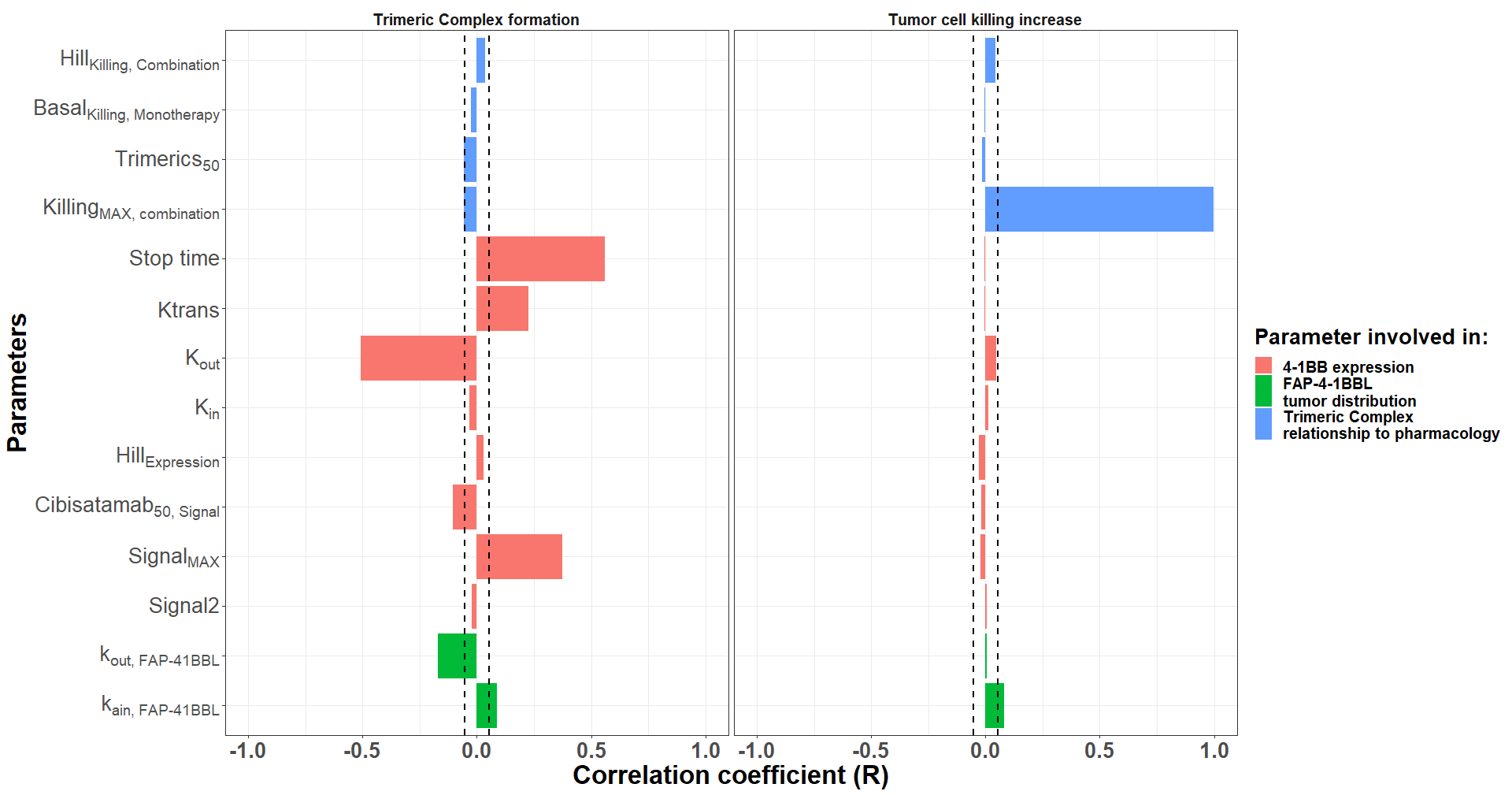
Figure S3. Sensitivity analysis of the model. Color code indicates what each parameter is involved in. Lognormal uncertainty distributions (consisting of 1,000 parameter values) were created for each parameter using the estimated relative standard error as standard deviation of the random effects. For tumor distribution parameters (green), which are fixed in the original model, the relative standard error was fixed to 40%. FAP-4-1BBL was administered at the dose maximizing trimeric complex formation over a period of 120 hours. For simplicity, PK parameters are omitted from the analysis. The correlation coefficient between parameter values and average trimeric complex formation or average increase in tumor cell killing was used as a metric of model sensitivity to parameter values regardless of the magnitude of change.*
